# Large plasmidome of dairy *Lactococcus lactis* subsp. *lactis* biovar diacetylactis FM03P encodes technological functions and appears highly unstable

**DOI:** 10.1186/s12864-018-5005-2

**Published:** 2018-08-17

**Authors:** Oscar van Mastrigt, Elisa Di Stefano, Sylviani Hartono, Tjakko Abee, Eddy J. Smid

**Affiliations:** Food Microbiology, Wageningen University & Research, P.O. Box 17, 6700AA, Wageningen, The Netherlands

**Keywords:** Lactic acid bacteria, Dairy, Plasmid, Protoplast-induced curing, Functionality, Stability

## Abstract

**Background:**

Important industrial traits have been linked to plasmids in *Lactococcus lactis*.

**Results:**

The dairy isolate *L. lactis* subsp. *lactis* biovar diacetylactis FM03P was sequenced revealing the biggest plasmidome of all completely sequenced and published *L. lactis* strains up till now. The 12 plasmids that were identified are: pLd1 (8277 bp), pLd2 (15,218 bp), pLd3 (4242 bp), pLd4 (12,005 bp), pLd5 (7521 bp), pLd6 (3363 bp), pLd7 (30,274 bp), pLd8 (47,015 bp), pLd9 (15,313 bp), pLd10 (39,563 bp), pLd11 (9833 bp) and pLd12 (3321 bp). Structural analysis of the *repB* promoters and the RepB proteins showed that eleven of the plasmids replicate via the theta-type mechanism, while only plasmid pLd3 replicates via a rolling-circle replication mechanism. Plasmids pLd2, pLd7 and pLd10 contain a highly similar operon involved in mobilisation of the plasmids. Examination of the twelve plasmids of *L. lactis* FM03P showed that 10 of the plasmids carry putative genes known to be important for growth and survival in the dairy environment. These genes encode technological functions such as lactose utilisation (*lacR-lacABCDFEGX*)*,* citrate uptake (*citQRP*), peptide degradation (*pepO* and *pepE*) and oligopeptide uptake (*oppDFBCA*), uptake of magnesium and manganese (2 *mntH*, *corA*), exopolysaccharides production (*eps* operon), bacteriophage resistance (1 *hsdM*, 1 *hsdR* and 7 different *hsdS* genes of a type I restriction-modification system, an operon of three genes encoding a putative type II restriction-modification system and an abortive infection gene) and stress resistance (2 *uspA*, *cspC* and *cadCA*). Acquisition of these plasmids most likely facilitated the adaptation of the recipient strain to the dairy environment. Some plasmids were already lost during a single propagation step signifying their instability in the absence of a selective pressure.

**Conclusions:**

*Lactococcus lactis* FM03P carries 12 plasmids important for its adaptation to the dairy environment. Some of the plasmids were easily lost demonstrating that propagation outside the dairy environment should be minimised when studying dairy isolates of *L. lactis*.

**Electronic supplementary material:**

The online version of this article (10.1186/s12864-018-5005-2) contains supplementary material, which is available to authorized users.

## Background

*Lactococcus lactis* is a lactic acid bacterium which is extensively used in food fermentation processes. It is one of the main species used in starter cultures for the production of fermented dairy products, such as cheese, quark, cottage cheese and sour cream [1, 2]. *L. lactis* is naturally present on plants [3, 4] and it is proposed that dairy strains have evolved from plant-associated strains transferred to milk via cattle [1, 5–10]. *L. lactis* has adapted to the dairy environment by the acquisition of important traits required for the growth on milk, such as lactose catabolism, proteinase activity, citrate utilisation and bacteriophage resistance. Analysis of genomes of *L. lactis* has shown that these traits are often encoded by genes located on plasmids [11].

Plasmids are mobile, self-replicating extrachromosomal DNA molecules which can be lost and acquired in response to changing environmental conditions. This behaviour facilitates their distribution among bacteria occupying the same ecological niche. Depending on the environmental conditions, plasmids could have beneficial or adverse effects for the recipient strain. They could give the bacteria the ability to grow better on particular nutrients or survive better under harsh conditions, but at the same time they can be a metabolic burden by either replication of the plasmids or by expression of the plasmid-encoded genes [12].

In this study, the complete nucleotide sequences of twelve plasmids of the dairy isolate of *L. lactis* subsp. *lactis* biovar diacetylactis FM03P are presented, together with analysis of the putative biological functions that were assigned to them. Plasmid-cured variants were made by protoplast-induced curing to confirm some of the putative functions and to demonstrate the impact of the plasmids on growth.

## Results and discussion

### Sequencing

*Lactococcus lactis* subsp. *lactis* biovar diacetylactis FM03P has been isolated from 10-week-old Samsø cheese. Its genome has been sequenced using a combination of an Illumina HiSeq2500 and PacBio RS instrument as previously described [13]. This revealed the complete sequence of the chromosome and 7 plasmids, designated pLd1 to pLd7. Subsequent next-generation sequencing attempts of *L. lactis* FM03-V1, a single colony isolate of a culture of *L. lactis* FM03P, using only Illumina revealed the sequence of 5 other plasmids, designated pLd8 to pLd12. The different sequencing attempts have been summarised in (Additional file 1: Figure S1). Using PCR and analysis by gel electrophoresis, we confirmed the presence of all 12 plasmids in parent strain *L. lactis* FM03P (Additional file 1: Figure S2). The obtained sequences of pLd8 to pLd12 were not present in the raw unassembled PacBio and Illumina reads obtained from strain FM03P demonstrating that these plasmids were already lost during propagation.

**Fig. 1:**
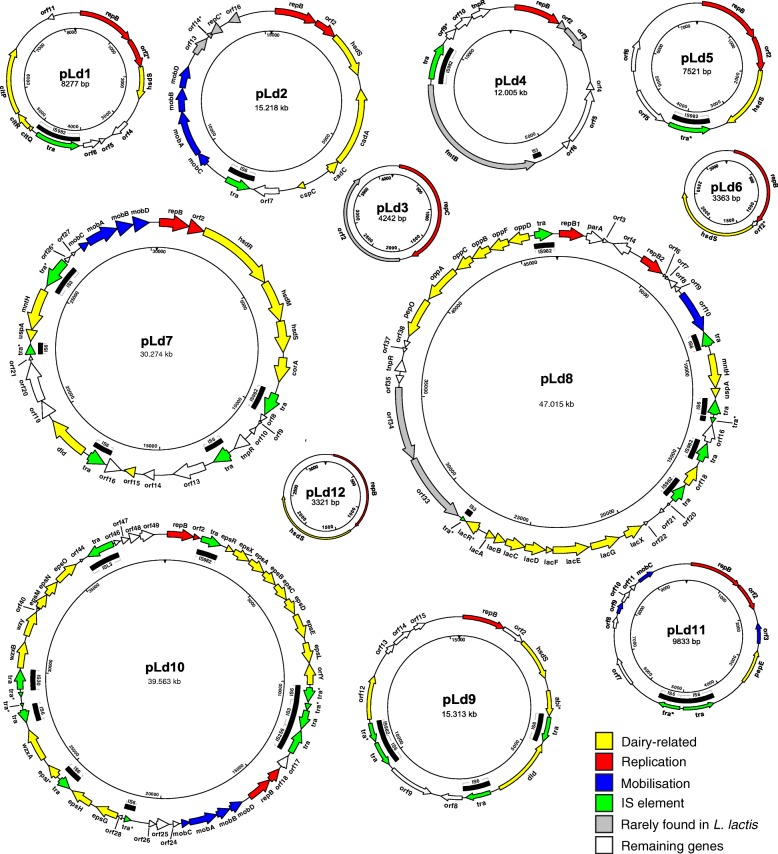
Genetic maps of plasmids of *L. lactis* FM03P. Arrows indicate positions and direction of predicted genes. Colours represent the putative functions of the genes. The name of the genes are indicated and correspond to gene names used in Table 2. Pseudogenes are marked with an asterisk. The inner circle corresponds to the nucleotide numbering of the plasmid

The genome of *L. lactis* subsp. *lactis* biovar diacetylactis FM03P contains a chromosome of 2.43 Mbp with a G + C content of 35.3% and 12 plasmids with sizes of 8.3, 15.2, 4.2, 12.0, 7.5, 3.4, 30.3, 47.0, 15.3, 39.6, 9.8, and 3.3 kbp and G + C contents of 34.8, 34.1, 35.6, 33.5, 33.6, 33.8, 35.2, 35.3, 35.2, 34.9, 33.0, and 33.2%, respectively (Fig. 1). The obtained plasmid sequences were annotated using RAST [14] after which the annotation was manually curated and analysed in detail including their replication mechanisms and mobilisation properties.

### Replication

Plasmids replicate independently from the chromosome using plasmid-encoded Rep proteins. Plasmids in lactococci replicate using either rolling-circle replication (RCR) or theta-type replication [15]. The replication mechanism as well as the sequence of the replication protein and the origin of replication affect the stability of the plasmids, their copy number and their compatibility. RCR plasmids replicate via the synthesis of ssDNA intermediates, and these plasmids are usually small in size, have multiple copies and are incompatible with other RCR plasmids [16, 17]. Sequence homology of the replication initiator protein and the presence of a double-stranded origin of replication (*dso*) indicated that pLd3 is the only RCR plasmid in *L. lactis* FM03P. On pLd2 we found a gene encoding a protein that had high similarity to the N-terminal part of RepB which is normally encoded on RCR plasmids of the type pMV158, but the C-terminal end was found to be missing.

Theta plasmids are more common in lactococci and a single bacterial cell can contain multiple theta plasmids [18]. Based on the homology of the replication initiator protein and structural motifs in the *repB* promoter, it was concluded that all plasmids except pLd3 replicate with a theta-type mechanism (Additional file 1: Figures S3 and S4). On plasmid pLd8 two *repB* genes are located of which the second replicon (with the *repB2* gene) seems to be functional as it is highly similar to the replicons of other plasmids. In contrast, in the promoter of *repB1* the inverted repeat IRb is missing and the C-terminus of the predicted RepB1 protein is quite different from the other predicted RepB proteins (including RepB2). Both the *repB* genes on pLd10 seem to be functional. The DNA sequences of the *repB* genes on plasmids pLd2 and pLd9 and their promoters are 100% identical. This could cause incompatibility of these plasmids and could result in plasmid loss [15, 19]. This may also explain why plasmid pLd9 was not found in the first sequencing attempt. We also observed spontaneous loss of plasmid pLd2 during continuous chemostat cultivation, while pLd9 was kept (data not shown).

### Mobilisation

**Fig. 2:**
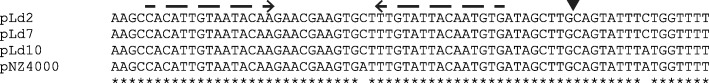
Multiple sequence alignment of *oriT* regions of plasmids pLd2, pLd7, pLd10 and pNZ4000 [21]. The inverted repeat is shown with dashed arrows and the arrowhead indicates the *nic* site. The asterisks below indicate identical nucleotides in all four sequences

Plasmids can carry conjugation or mobilisation regions that increase their spread in the population via conjugation events. Conjugative plasmids are self-transmissible, while mobilisable plasmids are only transmissible in the presence of additional conjugative functions [20]. To determine if plasmids were transmissible, the plasmid sequences were searched for known conjugation and mobilisation regions. Plasmids pLd2, pLd7 and pLd10 showed a highly similar (> 97%) operon of 4 genes involved in mobilisation of the plasmid. The DNA sequence of these operons in pLd2, pLd7 and pLd10 is 96, 97 and 98% similar to the mobilisation region of pNZ4000, respectively, which was demonstrated to be functional [21]. Plasmid pNZ4000 was found in *Lactococcus lactis* and carries genes necessary for the production of exopolysaccharides. The *oriT* sequences, essential for plasmid mobilisation, of pLd2, pLd7 and pLd10 are highly similar to the functional *oriT1* sequence of pNZ4000 (Fig. 2) and all plasmids carry a *mobA* gene encoding a relaxase that is involved in nicking at the *nic* sites of the *oriT* sequences. Plasmids pLd2, pLd7 and pLd10 also carry *mobC* which is present but not annotated in pNZ4000. The genes *mobC* and *mobB*, of which the start codon overlaps with the stop codon of *mobA¸* most likely encode accessory proteins for MobA [22, 23]. The function of *mobD*, designated *mobC* in pNZ4000, remains to be elucidated. Recently, the nucleotide sequence of plasmid p229C of *L. lactis* 229 has been published [24]. This plasmid is remarkably similar to plasmid pLd7 (> 99.9%), while the other plasmids show no or limited similarity. Both strains are isolated from the dairy environment and the mobilisation operon might have increased the transfer rate of this plasmid within the dairy environment. In addition to the mobilisation operons, pLd11 carries 3 genes (*orf3, orf9 and mobC*) encoding proteins which may be involved in mobilisation. Moreover, pLd8 carries a gene encoding a putative conjugal transfer protein (nickase) of the MobA-MobL family. In contrast to the described genes on pLd2, pLd7 and pLd10, these genes are not part of a large mobilisation gene cluster.

### Plasmid-encoded functions

**Fig. 3:**
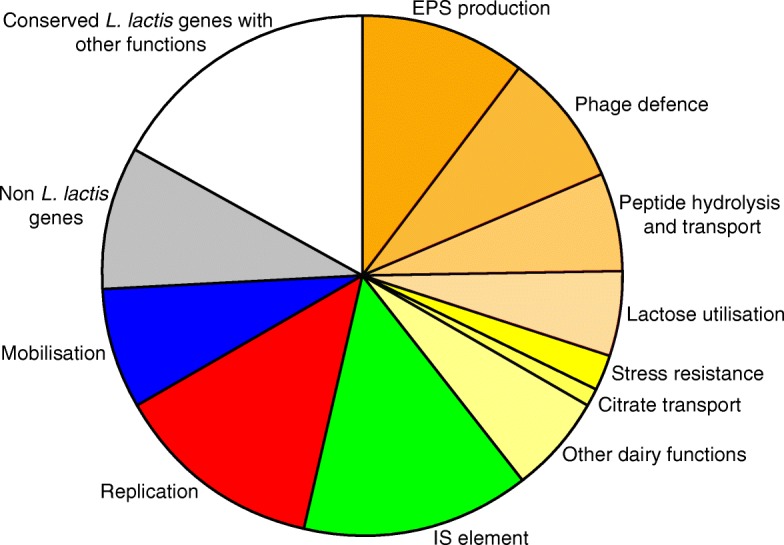
Overview of the putative functions of plasmid-encoded genes in *L. lactis* FM03P. The sum of the sizes of the genes within a category relative to the total size of all plasmid genes was used. The orange to yellow categories represent dairy functions. Other dairy functions include genes for uptake of magnesium and manganese (*corA* and *mntH*), D-lactate dehydrogenases (*dld*), and a C4-dicarboxylate transporter. Non *L. lactis* genes include the genes that are rarely found in *L. lactis*. Conserved *L. lactis* genes with other functions consist of all the putative genes which are regularly found in *L. lactis* and encode hypothetical proteins or proteins with functions that did not fit into the other categories

On the 12 plasmids, we identified and annotated in total 203 putative genes or fragments thereof, which corresponds to 7.4% of the total number of putative genes present in the entire genome (Table 1). Based on homology with other proteins, putative functions were ascribed to 74% of the plasmid encoded genes. Of all genes 10% were pseudogenes containing frameshifts, a premature stop codon or truncations. An overview of all the genes in the plasmidome of *L. lactis* FM03P is given in Fig. 3 and their putative functions are given in Table 2. In the coming sections a selection of genes encoding functions that could enhance growth and survival of the bacteria and/or encode technological properties are described in more detail.

**Table 1:** Summary of plasmid statistics and the putative genes

	Size (bp)	G + C content (%)	Total no. of:		
			ORFs	Pseudogenes^a^	Homolog with known function
pLd1	8277	33.8	11	1 (0)	7
pLd2	15,218	35.6	16	3 (0)	14
pLd3	4242	33.6	2	0	1
pLd4	12,005	34.8	11	1	4
pLd5	7521	33.5	6	0	5
pLd6	3363	34.1	3	1	2
pLd7	30,274	35.2	31	3 (2)	23
pLd8	47,015	35.3	45	3 (2)	32
pLd9	15,313	35.2	15	2 (1)	12
pLd10	39,563	34.9	49	5 (4)	40
pLd11	9833	33.0	12	1 (1)	8
pLd12	3321	33.2	2	0	2
Total	195,945		203	20 (10)	150

^a^Values in parentheses indicate number of pseudogenes in IS elements

**Table 2:** Overview of the putative genes and their putative functions

Plasmid	Gene	Putative function	Best homolog found in:
pLd1	*repB*	Replication initiator protein	
	orf2*	Replication-associated protein	
	*hsdS*	**Type I restriction-modification system specificity subunit S**	
	*orf4*	Hypothetical protein	
	*orf5*	Hypothetical protein	
	*orf6*	Hypothetical protein	
	*tra*	Transposase IS*982* family	
	*citQ*	**Leader peptide CitQ**	
	*citR*	**Translational regulator**	
	*citP*	**Citrate transporter**	
	*orf11*	Hypothetical protein	
pLd2	*repB*	Replication initiator protein	
	orf2*	Replication-associated protein	
	*hsdS*	**Type I restriction-modification system specificity subunit S**	
	*cadA*	**Cadmium-transporting ATPase**	
	*cadC*	**Cadmium efflux system accessory protein**	
	*cspC*	**Cold shock protein**	
	*orf7*	Serine/threonine protein phosphatase	
	*tra*	Transposase IS*6* family	
	*mobC*	Mobilisation protein	
	*mobA*	Mobilisation protein	
	*mobB*	Mobilisation protein	
	*mobD*	Mobilisation protein	
	*orf13*	HXXEE domain-containing protein	*Streptococcus thermophilus*
	orf14*	XRE family transcriptional regulator	*Streptococcus thermophilus*
	repC*	Replication initiator protein	*Lactobacillus farciminis*
	*orf16*	Hypothetical protein	*Enterococcus faecium*
pLd3	*repC*	Replication initiator protein	
	*orf2*	Hypothetical protein	*Lactobacillus reuteri*
pLd4	*repB*	Replication initiator protein	
	*orf2*	Hypothetical protein	*Enterococcus faecalis*
	*orf3*	Hypothetical protein	*Enterococcus faecalis*
	*orf4*	Hypothetical protein	
	*orf5*	Hypothetical protein	
	*orf6*	Hypothetical protein	
	*fmtB*	Peptidoglycan-binding protein	*Streptococcus thermophilus*
	*tra*	Transposase IS*982* family	
	orf9*	DNA-directed DNA polymerase	
	*orf10*	Hypothetical protein	
	*tnpR*	Resolvase	
pLd5	*repB*	Replication initiator protein	
	*orf2*	Replication-associated protein	
	*hsdS*	**Type I restriction-modification system specificity subunit S**	
	*tra*	Transposase IS*982* family	
	*orf5*	Hypothetical protein	
	*orf6*	Site-specific integrase	
pLd6	*repB*	Replication initiator protein	
	orf2*	Hypothetical protein	
	*hsdS*	**Type I restriction-modification system specificity subunit S**	
pLd7	*repB*	Replication initiator protein	
	*orf2*	Replication-associated protein	
	*hsdR*	**Type I restriction-modification system specificity subunit R**	
	*hsdM*	**Type I restriction-modification system specificity subunit M**	
	*hsdS*	**Type I restriction-modification system specificity subunit S**	
	*corA*	**Magnesium transporter**	
	*tra*	Transposase IS*982* family	
	*orf8*	Hypothetical protein	
	*orf9*	Hypothetical protein	
	*orf10*	Hypothetical protein	
	*tnpR*	Resolvase	
	*tra*	Transposase IS*6* family	
	*orf13*	MFS transporter	
	*orf14*	Acetyltransferase	
	*orf15*	**Polysaccharide biosynthesis protein**	
	*orf16*	Hypothetical protein	
	*tra*	Transposase IS*6* family	
	*dld*	**D-Lactate dehydrogenase**	
	*orf19*	Hypothetical protein	
	*orf20*	Hypothetical protein	
	*orf21*	Hypothetical protein	
	tra*	Transposase IS*6* family	
	*uspA*	**Universal stress protein**	
	*mntH*	**Manganese transporter**	
	tra*	Transposase IS*3* family	
	orf26*	integrase/recombinase	
	*orf27*	Hypothetical protein	
	*mobC*	Mobilisation protein	
	*mobA*	Mobilisation protein	
	*mobB*	Mobilisation protein	
	*mobD*	Mobilisation protein	
pLd8	*repB1*	Replication initiator protein	
	*parA*	Chromosome partitioning protein	
	*orf3*	Hypothetical protein	
	*orf4*	Serine protease	
	*repB2*	Replication initiator protein	
	*orf6*	Hypothetical protein	
	*orf7*	Hypothetical protein	
	*orf8*	Hypothetical protein	
	*orf9*	Hypothetical protein	
	*orf10*	Nickase	
	*tra*	Transposase IS*6* family	
	*mntH*	**Manganese transporter**	
	*uspA*	**Universal stress protein**	
	*tra*	Transposase IS*6* family	
	tra*	Transposase IS*6* family	
	*orf16*	AAC(3) family N-acetyltransferase	
	*tra*	Transposase IS*982* family	
	*orf18*	**C4-dicarboxylate ABC transporter**	
	*tra*	Transposase IS*982* family	
	*orf20*	Hypothetical protein	
	*orf21*	Hypothetical protein	
	*orf22*	**Large-conductance mechanosensitive channel**	
	*lacX*	Hypothetical protein	
	*lacG*	**6-phospho-beta-galactosidase**	
	*lacE*	**PTS lactose transporter subunit IIBC**	
	*lacF*	**PTS lactose transporter subunit IIA**	
	*lacD*	**Tagatose 1,6-diphosphate aldolase**	
	*lacC*	**Tagatose-6-phosphate kinase**	
	*lacB*	**Galactose-6-phosphate isomerase subunit LacB**	
	*lacA*	**Galactose-6-phosphate isomerase subunit LacA**	
	lacR*	**Lactose repressor**	
	tra*	Transposase IS*3* family	
	*orf33*	**Type II restriction modification system**	*Leuconostoc mesenteroides*
	*orf34*	**Type II restriction modification system**	*Leuconostoc mesenteroides*
	*orf35*	Hypothetical protein	
	*tnpR*	Resolvase	
	*orf37*	Hypothetical protein	
	*orf38*	Hypothetical protein	
	*pepO*	**Neutral endopeptidase**	
	*oppA*	**Peptide-binding protein**	
	*oppC*	**Peptide ABC transporter permease**	
	*oppB*	**Peptide ABC transporter permease**	
	*oppF*	**Oligopeptide transport ATP-binding protein**	
	*oppD*	**Oligopeptide transport ATP-binding protein**	
	*tra*	Transposase IS*982* family	
pLd9	*repB*	Replication initiator protein	
	*orf2*	Hypothetical protein	
	*hsdS*	**Type I restriction-modification system specificity subunit S**	
	abi*	**Abortive phage resistance protein**	
	*tra*	Transposase IS*6* family	
	*dld*	**D-lactate dehydrogenase**	
	*tra*	Transposase IS*6* family	
	*orf8*	Hypothetical protein	
	*orf9*	MFS transporter	
	*tra*	Transposase IS*6* family	
	tra*	Transposase IS*982* family	
	*orf12*	Amidohydrolase of peptidase M20 family	
	*orf13*	Hypothetical protein	
	*orf14*	Site-specific integrase	
	*orf15*	Integrase-associated protein	
pLd10	*repB1*	Replication initiator protein	
	*orf2*	Replication-associated protein	
	*tra*	Transposase IS*982* family	
	*epsR*	**XRE family transcriptional regulator**	
	*epsX*	**Polysaccharide biosynthesis protein**	
	*epsA*	**Tyrosine protein kinase transmembrane modulator**	
	*epsB*	**Tyrosine protein kinase**	
	*epsC*	**Tyrosine protein phosphatase**	
	*epsD*	**Undecaprenyl-phosphate galactosephosphotransferase**	
	*epsE*	**Group 1 glycosyltransferase**	
	*epsL*	**Exopolysaccharide biosynthesis protein**	
	*orfY*	**LytR family transcriptional regulator**	
	tra*	Transposase IS*6* family	
	*tra*	Transposase IS*3* family	
	*tra*	Transposase IS*3* family	
	*tra*	Transposase IS*256* family	
	*orf17*	XRE family transcriptional regulator	
	*orf18*	Replication-associated protein	
	*repB2*	Replication initiator protein	
	*mobD*	Mobilisation protein	
	*mobB*	Mobilisation protein	
	*mobA*	Mobilisation protein	
	*mobC*	Mobilisation protein	
	*orf24*	Hypothetical protein	
	*orf25*	Integrase	
	*orf26*	Hypothetical protein	
	tra*	Transposase IS6 family	
	*orf28*	Hypothetical protein	
	*epsG*	**Glycosyltransferase family 2 protein**	
	*epsH*	**Glycosyltransferase family 2 protein**	
	*tra*	Transposase IS*6* family	
	epsI*	**Glycosyltransferase**	
	*wzxA*	**Flippase**	
	*tra*	Transposase IS*6* family	
	tra*	Transposase	*Lactobacillus/Oenococcus/Enterococcus*
	tra*	Transposase	*Lactobacillus*
	*tra*	Transposase IS*30* family	*Lactobacillus*
	*wzxB*	**Flippase**	*Lactobacillus*
	*wzy*	**Polymerase**	*Lactobacillus*
	*orf40*	Hypothetical protein	
	*epsM*	**Glycosyltransferase family 2 protein**	*Lactobacillus*
	*epsN*	**Glycosyltransferase**	*Lactobacillus*
	*epsO*	**Glycosyltransferase family 1 protein**	*Lactobacillus*
	*orf44*	DUF4411 domain-containing protein	*Lactobacillus plantarum*
	*tra*	Transposase IS*L3* family	*Lactobacillus*
	*orf46*	Hypothetical protein	*Lactobacillus*
	*orf47*	Hypothetical protein	
	*orf48*	Hypothetical protein	
	*orf49*	Resolvase	
pLd11	*repB*	Replication initiator protein	
	*orf2*	Replication-associated protein	
	*orf3*	Relaxase/mobilisation nuclease domain protein	
	*pepE*	**Peptidase E**	
	*tra*	Transposase IS*6* family	
	tra*	Transposase IS*6* family	
	*orf7*	DUF1919 domain-containing protein	
	*orf8*	DUF3883 domain-containing protein	
	*orf9*	Relaxase	
	*orf10*	Hypothetical protein	
	*orf11*	Hypothetical protein	
	*mobC*	Mobilisation relaxosome protein	
pLd12	*repB*	Replication initiator protein	
	*hsdS*	**Type I restriction-modification system specificity subunit S**	

Putative functions that could be beneficial in the dairy environment are shown in bold. Pseudogenes are indicated with an asterisk. The species that contains the best homolog is given of genes that are rarely found in *L. lactis*

### Substrate uptake and utilisation

To thrive in particular environments, microorganisms require specific transporters and metabolic pathways to take up substrates from the environment and use them for growth. Therefore, the presence of genes encoding particular transporters or metabolic enzymes indicate adaptation to a specific environments. To investigate if *L. lactis* FM03P was adapted to the dairy environment, we searched the plasmid sequences for genes involved in utilisation of the main carbon and energy sources found in bovine milk: citrate, lactose and proteins.

Citrate utilisation is characteristic for the biovariety diacetylactis of *Lactococcus lactis* that contains a plasmid-encoded *citQRP* operon. In *L. lactis* FM03P, this operon is located on plasmid pLd1, which is 99% identical to lactococcal plasmids pCRL1127 and pIL2. The *citP* gene is encoding a citrate permease enabling the host to take up divalent citrate from the environment [25]. Citrate utilisation results in the generation of a proton motive force in *L. lactis* [26] and at the same time increases the pH of the environment [27]. Citrate utilisation also has been linked to the production of acetoin and diacetyl. These buttery aromas are important flavour compounds in dairy products. Both *citQ* and *citR* are involved in the regulation of expression if *citP* [28, 29]. The gene *citP* is mainly expressed at low pH (around 5.5) when the abundance of divalent citrate is maximal [30, 31], minimising the metabolic burden of maintaining this plasmid.

Genes involved in lactose uptake and utilisation are found on plasmid pLd8, which contains the *lacR-lacABCDFEGX* operon for lactose uptake via a phosphotransferase system (PTS) and utilisation via the tagatose-6-phosphate pathway. As described for *L. lactis* IL1403 [32], *L. lactis* FM03P also contains the chromosomal-encoded Leloir pathway for lactose utilisation. The presence of both pathways in one strain might give this strain a competitive advantage by fast uptake and utilisation of lactose. Interestingly, the LacR protein might not be functional due to a 40 amino acids deletion at the C-terminus, most likely caused by an IS element insertion in the *lacR* gene. LacR is a transcriptional repressor of the *lac* operon and deletion of the *lacR* gene has been shown to increase the activity of the *lac* promoter both during growth on glucose and lactose [33, 34]. Therefore, a non-functional LacR might increase the maximum lactose utilisation rate.

Genes involved in utilisation of proteins, or more specifically oligopeptides, are found on plasmid pLd8, which contains the *pepO* gene encoding a neutral endopeptidase and the complete *oppDFBCA* gene cluster encoding the oligopeptide permease (Opp) system [35]. All of these genes are also encoded on the chromosome with a high similarity in amino acid sequence (> 99%), except for *oppA* which only has 87% similarity to its chromosomal homolog. Plasmid pLd11 carries a *pepE* gene encoding a putative aspartyl dipeptidase E, which does not have a chromosomal homolog. The *opp* and *pep* genes are required, in combination with the extracellular protease PrtP, for utilisation of the milk caseins as nitrogen source [35, 36]. The extracellular protease is often plasmid encoded by *prtP* and *prtM* [37]. Interestingly, the *prtP* and *prtM* genes are not found in *L. lactis* FM03P and this strain does not show a caseinolytic phenotype (data not shown). Presence of the *pepE*, *pepO* and *oppDFBCA* gene cluster and absence of the *prtP* and *prtM* genes could give the strain an advantage when growing in combination with a protease-positive (prt^+^) strain in milk, in particular at high cell densities [38]. Only the prt^+^ strain secretes the protease to hydrolyse the milk caseins, thereby investing energy in production of this protein, while the protease-negative (prt^−^) strain can use the peptides generated by the protease without having the burden of protease expression. This combination of prt^+^ and prt^−^ strains is found in many dairy starter cultures, for instance in the Ur starter culture [39]. The presence of *pepO* and *opp* genes on both the chromosome and plasmid pLd8 could result in faster utilisation of the peptides depending on the copy number of the plasmid and the regulation of expression of the *pepO* and *opp* genes.

### Phage resistance by restriction-modification systems

**Fig. 4:**
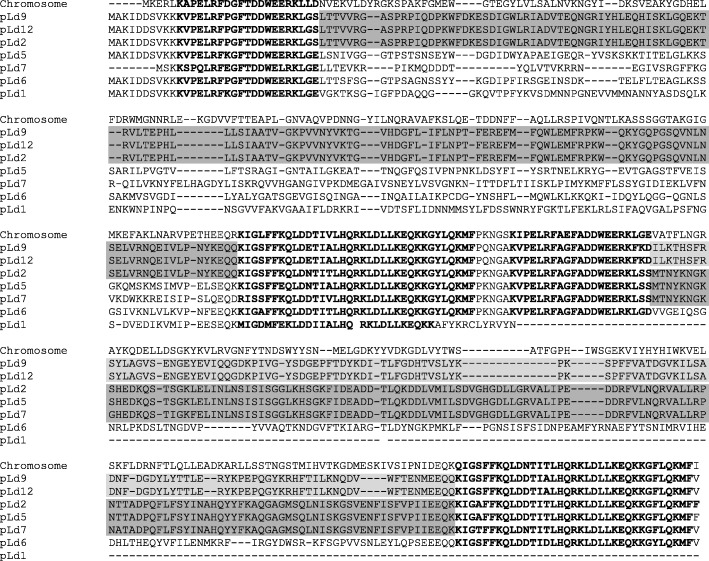
Alignment of predicted amino acid sequences of HsdS proteins encoded by genes of *Lactococcus lactis* FM03P. The conserved regions are shown with boldface letters [43]. Shaded boxed show the identical amino acids sequence in the variable regions of different HsdS proteins

An important technological property that is often carried by plasmids is the resistance to bacteriophage infections via the type I restriction-modification system comprising of three subunits. HsdS and HsdM are both necessary for methyltransferase activity, while HsdR is required in addition to the HsdS-HsdM complex for restriction of foreign’ DNA that is not methylated [40]. The HsdS subunit contains two variable target recognition domains (TRDs) that determine the target sequence specificity of both the restriction and modification activities of the complex [40, 41]. The variable domains are flanked by conserved regions required for specific associations with the other subunits and for maintaining the relative positions of the two TRDs [40]. In *L. lactis* FM03P the three subunits of this system (HsdR, HsdM and HsdS) are all encoded once on the chromosome. On 7 out of the 12 plasmids other HsdS subunits are found. The protein sequences of all HsdS proteins were aligned with MAFFT (Fig. 4). Most of the HsdS proteins contained two variable regions flanked by highly conserved regions as expected. However, the HsdS protein encoded on plasmid pLd1 is not complete. The *hsdS* gene carried by pLd1 is about half of the size, most likely due to a deletion of 500 to 600 nucleotides. Interestingly, the second variable region of the HsdS proteins of pLd2 and pLd5 are identical and also 97% similar to pLd7, while the first variable regions do not show significant homology to each other. Similarly, the first variable regions of the HsdS proteins of pLd2, pLd9 and pLd12 are identical, while the second variable region of pLd2 does not show significant homology to those of pLd9 and pLd12. The high similarities of one of the variable regions suggest homologous recombination events as also found for plasmids pAH33 and pAH82 [42]. This could result in new R/M specificities.

In addition to the many *hsdS* genes, on pLd7 also HsdR and HsdM subunits are encoded. These proteins have a low homology to their chromosomal counterparts (41% and 34%, respectively), although the C-terminus of HsdM is quite similar to its chromosomal counterpart. As Schouler and co-authors [43] described before, the C-terminal parts of the HsdM subunits of different families have a common sequence that could be involved in the association of HsdM with HsdS. Therefore, the different HsdM and HsdR variants found on the chromosome and the plasmids in combination with the many different plasmid encoded HsdS subunits can form an effective recombination system for broadening the target specificity of the system.

In addition to the type I R/M system, plasmid pLd8 harbours *orf33, orf34* and *orf35* encoding a putative type II restriction-modification system. A similar operon is found in *Leuconostoc mesenteroides* LK-151 (90%) and in the strains JM3, SK110 and N41 of *Lactococcus lactis*. *orf34* is predicted to encode a type IIG restriction enzyme/N6-adenine DNA methyltransferase according to the restriction enzymes database REBASE [44], which contains both a methylase and recognition domain (pfam12950) according to the SMART database [45]. However, no restriction domain was found in the protein encoded by *orf34*. The restriction domain is most likely part of the protein encoded by *orf33*. This protein contains a putative phospholipase D (PLD) domain (pfam13091), a SNF2 ATPase domain (pfam00176), a DEAD-like helicase domain and a helicase C-terminal domain (pfam00271) according to the SMART database. The PLD domain is the metal-independent catalytic site in type IIS restriction endonucleases [46] and might also catalyse the restriction. Notably, the predicted domains in the proteins encoded by *orf33* and *orf34* are similar to the domains found in the recently identified Class I DISARM system for bacteriophage defence in which the methylase was also predicted to be of the IIG type [47], although they do not share a similar organisation.

Finally, plasmid pLd9 carries an *abi* gene encoding a putative abortive infection system. The first 642 nucleotides of this gene are 100% identical to a gene encoding the characterised abortive infection system Abi-859 [48]. However, the last 207 nucleotides are missing in pLd9 due to an insertion of a mobile element, so this system might not be functional.

### Exopolysaccharide (EPS) production

**Fig. 5:**
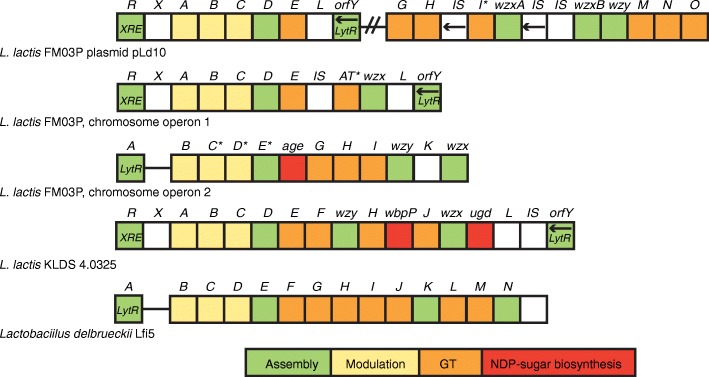
Schematic genetic organisation of the exopolysaccharide (EPS) gene clusters in *L. lactis* FM03P. Typical *eps* gene clusters of *L. lactis* and *Lactobacillus delbrueckii* are shown [50]. Colours represent different functional groups. Genes with unknown functions or functions not related to EPS biosynthesis are shown in white. All genes are transcribed in the forward direction except for a few genes oriented in the opposite direction, marked with arrows. Pseudogenes are marked with an asterisk. *XRE* and *LytR* represent the type of the transcriptional regulator. The letter and names above the boxed represent the gene names, in which only the last letter of the gene name is given for genes starting with *eps*. The ‘GT’ functional module consists of glycosyltransferases and other enzymes modifying the repeat unit structure, like the acetyltransferase (AT). This figure is adapted from [50]. IS: transposase; NDP-sugar: nucleotide diphopho-sugar

Exopolysaccharides are thought to have several functions including protection against low-moisture environments and toxic compounds (e.g. bile salts and hydrolysing enzymes, metal ions and antibiotics), colonisation and preventing phage infection [49]. However, in the dairy industry the most important function of EPS is that due to its water-binding capacity, it may improve the rheological properties of the fermented product by affecting the viscosity, syneresis, firmness and taste perception [50]. Plasmid pLd10 carries 18 *eps* genes encoding putative proteins involved in polysaccharide production via the Wzy-dependent pathway. A typical *eps* gene cluster in *L. lactis* consists of the 6 highly conserved genes *epsR, epsX, epsA, epsB, epsC* and *epsD* at the 5′ end, a variable region including genes encoding a polymerase (*wxy*)¸ a flippase (*wzx*), one or more glycosyltranferases and/or other polymer-modifying enzymes and the conserved genes *epsL* and *orfY* at the 3′ end [50]. These genes are usually transcribed as a single mRNA [21]. Interestingly, in pLd10 the *eps* genes are distributed over two clusters and in between these clusters a replication and a mobilisation operon are located, which are flanked by IS elements (Fig. 1). The first cluster consists of: i) the *epsR* gene encoding an XRE family transcriptional regulator; ii) the conserved gene *epsX* with unknown function; iii) the phosphoregulatory module consisting of *epsABC*; iv) *epsD* encoding an undecaprenyl-phosphate galactosephosphotransferase which catalyses the first step in the assembly of the EPS basic repeating unit (i.e. addition of galactose-1-phosphate to the lipid-phosphate carrier); v) *epsE* encoding a putative glycosyltranferase; and finally (vi) *epsL* and *orfY* with an unknown and regulator function, respectively. The second cluster consists of 6 putative glycosyltransferase genes (*epsH, epsI, epsJ, epsO, epsP, epsQ*) of which *epsI* is most likely not functional, 2 putative flippases for export (*wzxA* and *wzxB*) and a putative polymerase (*wzy*). On the chromosome, two other *eps* clusters are found (Fig. 5). The first cluster has a typical organisation of *L. lactis eps* operons (*epsRXABCD – epsL-orfY)*, while the second cluster has a typical *Lactobacillus eps* cluster. This operon starts with *epsA* encoding a LytR transcriptional regulator followed directly by the phosphoregulatory module (*epsBCD*) and does not contain *epsX*, *epsL* and *orfY*.

### Cation transport

Cation transporters play an important role in growth and survival of microorganisms i) by protecting against toxic heave metals, like cadmium, ii) by providing essential metals, like magnesium, or iii) by providing metals that increase stress survival, like manganese. Several cation transporters were found on the plasmids of *L. lactis* FM03P.

Plasmid pLd2 carries genes encoding the proteins CadA and CadC, which are > 99.8 and 100% similar to CadA and CadC encoded on plasmids pAH82 of *L. lactis* DPC220 and pND302 of *L. lactis* M71 and on the chromosome of *Streptococcus thermophiles* 4134, respectively [51–53]. The *cadCA* genes have been shown to provide resistance towards cadmium and zinc in both *S. thermophiles* and *L. lactis* [51, 53]. CadC is a transcriptional repressor which binds to its own promoter region [54]. At high cadmium concentrations, CadC is released from its promoter resulting in the production of *cadCA* transcript and the expression of *cadA* [51]. CadA is an ATPase of the P-type catalysing the efflux of cadmium and zinc [55]. The gene *cadD*, which is located on the chromosome, also encodes a cadmium transporter with 44% similarity in amino acid sequence to the plasmid encoded (pRW001) CadD of *Staphylococcus aureus* [56].

On plasmid pLd7 we found the *corA* gene encoding a CorA family transporter, which is expected to be the main Mg^2+^ uptake system in bacteria but might transport other cations like cobalt or nickel as well [57]. The CorA protein on pLd7 is 99% identical to the CorA protein found on plasmid pSK11P of *L. lactis* SK11. On the chromosome three other CorA family transporters are found as well as two other Mg^2+^ transporting ATPases. The best match of all these proteins only had 27% homology to CorA of pLd7. Kehres et al. [58] observed a high degree of diversity in sequences within the CorA family even within one species and argued that members within the CorA family might have functions other than Mg^2+^ transport. This may also be the case for some of the CorA proteins found in the genome of *L. lactis* FM03P.

The third cation transporter on plasmids of *L. lactis* FM03P is the manganese transporter MntH, which belongs to the Nramp family of transporters for divalent metal ions. Mn^2+^ ion is an important trace metal required for growth and survival of many bacteria [59]. Several species of lactic acid bacteria accumulate Mn^2+^ to scavenge toxic oxygen species, especially superoxide radicals (**·**O_2_). This enables the bacteria to survive oxidative stress conditions [59]. Therefore, it is expected that the presence and activity of this transporter could enhance growth in environments with a low manganese concentration and increase survival towards oxidative stress. Plasmid pLd7 and pLd9 encode similar MntH proteins which are 99% homologous and belong to class C of the MntH proteins [60]. The chromosome of *L. lactis* FM03P harbours three operons that encode manganese transporters: i) a *mntH* gene of class Cβ, which contains a 344 bp deletion compared to the *mntH* Cβ gene of *L. lactis* subsp. *lactis* ATCC 11454 [60], ii) a *mntH* gene of class B with 99% identity to *mntH* B of *L. lactis* subsp. *lactis* ATCC 11454 [60], and iii) a *mtsBCA* operon encoding an ABC transporter for manganese. The *mntH* genes on the plasmids most likely take over the role of the disrupted chromosomal *mntH* Cβ gene.

### Stress resistance

Besides the potential oxidative stress resistance provided by MntH, there are universal stress proteins (UspA) encoded by genes on pLd7 and pLd9 and a cold shock protein CspC encoded on pLd2. This CspC protein is identical to the CspC protein of *L. lactis* MG1363 [61]. Furthermore, *orf22* carried by pLd8 encodes a putative large-conductance mechanosensitive channel which acts as osmotic release valve in response to a hypoosmotic shock preventing cell lysis [62].

### Antibiotic resistance

Plasmid pLd8 carries *orf16* encoding a putative aminoglycoside 3-N-acetyltransferase, which catalyses the acetylation of aminoglycoside antibiotics at the 3-amino group and thereby this gene is potentially involved in resistance towards these antibiotics [63]. BLAST analysis revealed highly similar genes (> 99% similarity), which are often found on plasmids in *L. lactis*, for instance in plasmids pJM3B, pSK11L, pC43 pJM2C, pUC06B, pJM4E, pUC109B, p158C, pCIS8, pJM1A and pUC08A. All these plasmid have in common that they are large (> 47 kb) and carry the *lacR-lacABCDFEGX* operon for lactose uptake and utilisation and the several oligopeptide transporters.

### Miscellaneous beneficial functions

Other functions encoded by genes on the plasmids that might be beneficial for the host are the putative FAD-dependent D-lactate dehydrogenases on pLd7 and pLd9, which are both 99% similar to the plasmid encoded putative D-lactate dehydrogenase from *L. lactis* SK11. Siezen et al. [64] suggested that D-lactate dehydrogenase could play a role in D-lactate utilisation in aerobic cultures, which could increase the external pH and the conversion to acetate leads to ATP production. However, Tanous et al. [65] did not find D-LDH activity in pGdh442-containing strains, which carry a highly similar *dld* gene, nor were they capable of growing on M17 containing D-lactate as carbon source. Plasmid pLd8 carries *orf18* encoding a C4-dicarboxylate ABC transporter, which function is to transport dicarboxylates such as aspartate, malate, fumarate, succinate and oxaloacetate. Finally, plasmid pLd7 and pLd9 both carry a gene encoding a transporter of the major facilitator superfamily.

### New genes

The plasmids found in *L. lactis* FM03P carry several genes that are rarely found before in *L. lactis*, which could indicate horizontal gene transfer events. BLASTP analysis of the amino acid sequence of the predicted protein encoded by *orf2* on plasmid pLd3 gave mainly hits from *Lactobacillus reuteri* (< 29% similar in amino acids) and contained a DUF3552 domain with unknown function. On pLd4 the *fmtB* gene, encoding a putative peptidoglycan-binding protein, has the highest homology to *Streptococcus thermophilus* strain B59671, although the homology only covers 60% of the gene. Both the *orf2* and the *fmtB* gene encode large proteins (569 and 1217 amino acids, respectively), account for a large fraction of the plasmids (40.3 and 30.4%, respectively), and are located on the only two plasmids which do not carry other genes with known dairy functions. The genes *orf33* and *orf34* on pLd8 were homologous to genes of *Leuconostoc mesenteroides* LK-151 encoding a putative type IIG restriction-modification system. Many genes in the *eps* operon on pLd10 (especially all genes from *tra* to *orf46*; Table 2) were most similar to *Lactobacillus* species and not found in *Lactococcus* species. Finally, various small (pseudo) genes were found which had the highest homology to species other than *L. lactis*. These include *orf13, orf14, repC* and *orf16* on pLd2, which are most similar (> 96%) to genes found in *Streptococcus, Lactobacillus* and *Enterococcus* and *orf2* and *orf3* on pLd4, which are similar to genes of *Enterococcus faecalis* (99 and 97%, respectively).

### Protoplast-induced curing

To determine the effect of plasmid content on the growth performance, plasmid-cured variants were made by protoplast-induced curing. After cells were harvested, the cell wall was degraded with lysozyme. Subsequently, protoplasts were regenerated on plates and the plasmid content of random selected colonies was analysed by PCR targeting genes on each plasmids followed by gel electrophoresis. The obtained variants are shown in Table 3.

**Table 3:** Variants of *Lactococcus lactis* FM03P with different plasmids contents that were obtained in this study

*L. lactis* variant	Cured plasmids	Plasmid content	Isolated from
FM03P	–	pLd1,2,3,4,5,6,7,8,9,10,11,12	Samsø cheese
3	pLd4,5,10	pLd1,2,3,6,7,8,9,11,12	Protoplast-induced curing
5	pLd4,10,11	pLd1,2,3,5,6,7,8,9,12	Protoplast-induced curing
47	pLd8,11	pLd1,2,3,4,5,6,7,9,10,12	Overnight culture
48	pLd2,4,5,6,7	pLd1,3,8,9,10,11,12	Chemostat culture
49	pLd2,3,4,5,6,7	pLd1,8,9,10,11,12	Protoplast-induced curing
50	pLd4,5,6,7	pLd1,2,3,8,9,10,11,12	Protoplast-induced curing
51	pLd4,5,6,7,8,9,11,12	pLd1,2,3,10	Protoplast-induced curing
63	pLd3,4,8,11,12	pLd1,2,5,6,7,9,10	Protoplast-induced curing
FM03-V1	pLd7	pLd1,2,3,4,5,6,8,9,10,11,12	Chemostat culture

Variants 47, 48 and FM03-V1 were isolated by plating on LM17 agar plates

### Plasmid loss in non-dairy environments

**Fig. 6:**
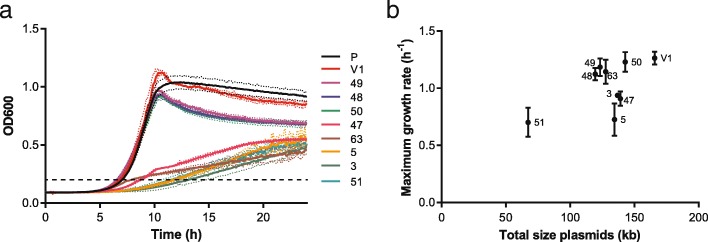
Growth of plasmid-cured variants of *L. lactis* FM03P in LM17. **a** Growth curves of the plasmid-cured variants. Dotted lines represent the standard deviation of representative biological duplicates. The dashed line represents an OD600 of 0.2, which was used as threshold to determine the time to detection. **b** Maximum growth rate as function of the total plasmid size calculated using the two-fold dilution method. Numbers near the symbols correspond to the different plasmid-cured variants. The maximum growth rate of the parent strain FM03P could not be estimated using the two-fold dilution method due to flocculation affecting the optical density measurement and thus the time to detection and is therefore omitted. Error bars represent the standard deviation of biological triplicates

The plasmids carry several genes important for growth in a dairy environment, but in non-dairy environments the plasmids have to be maintained with limited benefits and plasmid-cured variants might arise. Some plasmids were already lost during a single propagation step in either M17 supplemented with glucose or chemically-defined medium containing lactose showing that these plasmids were segregationally unstable in laboratory conditions in the absence of a selection pressure (e.g. bacteriophages, lactose, peptides) (Table 3). To determine the effect of the plasmid content on the growth of *L. lactis* in a non-dairy environment, the obtained plasmid-cured variants were grown in LM17 and the optical density at 600 nm was monitored. The growth curves significantly differed between the variants showing that the plasmid content did affect the growth in M17 (Fig. 6a). The highest maximum optical density was found for variant FM03-V1, which only lost plasmid pLd7 (OD 1.1). Variants 48, 49 and 50 had the same maximum growth rate (Fig. 6b) but reached a slightly lower maximum OD (between 0.91 and 0.96). These three variants all lost plasmids pLd4, pLd5 and pLd6, indicating that one of these plasmids might carry a gene which resulted in the higher OD. The growth curve of the parent strain FM03P was similar to variants 48, 59 and 50, but the maximum growth rate could not be estimated using the two-fold dilution method due to flocculation of this variant in M17. The remaining five variants reached a much lower maximum optical density and also grew slower (Fig. 6). Both variant 47 and 63 lost plasmid pLd8 carrying the *lacR-lacABCDFEGX* operon involved in lactose uptake and utilisation. Therefore, the drop in the growth rate in these variants at an optical density of approximately 0.25 could be caused by limited lactose utilisation and suggests that the *lacR-lacABCDFEGX* operon is functional when present. Variant 3, 5 and 51 were all growing much slower throughout the cultivation. Variant 3 and 5 are the only variants missing plasmid pLd10 carrying the *eps* genes, while variant 51 is the variant with the lowest number of plasmids since it has lost plasmids pLd4, pLd5, pLd6, pLd7, pLd8, pLd9, pLd11 and pLd12.

Additionally, growth performance of variants 48 and 50, the latter carrying plasmid pLd2 containing the *cadCA* operon, was determined in co-cultures in M17 supplemented with 0.5% glucose and 35 μM cadmium. Variant 50 dominated the populations after 27 generations (68%) which shows that pLd2 provided a competitive advantage in the presence of 35 μM cadmium. This is in line with the slightly higher maximal growth rates of variant 50 compared to that of variant 48 in the presence of cadmium (data not shown).

## Conclusions

In the past, important industrial traits have been linked to plasmids in *L. lactis*. Carrying twelve plasmids, *L. lactis* FM03P has the biggest plasmidome of all completely sequenced and published *L. lactis* strains up till now. Some of its plasmids were already lost during a single propagation step showing that the plasmids can be easily lost during propagation in a non-dairy environment. Because we directly sequenced the strain after it was isolated from cheese, the risk of plasmid loss was minimised. Examination of the twelve plasmids of *L. lactis* FM03P showed that 10 of the plasmids carry genes known to be important for the growth and survival in the dairy environment. These genes encode functions such as lactose and citrate utilisation, degradation and uptake of peptides, exopolysaccharide production, cation transport and bacteriophage and stress resistance. This shows that the plasmids play an important role in the adaptation of this strain to the dairy environment. Two plasmids, pLd3 and pLd4, did not carry any genes that are known to be linked to the dairy environment, but both do harbour a large gene with unknown function that has not been found before in *L. lactis* and may have a function relevant for dairy processing.

## Methods

### Strain and media

*Lactococcus lactis* subsp. *lactis* biovar diacetylactis FM03P, which has been isolated from 10-weeks-old Samsø cheese using Nickels and Leesment medium [66], was used in this study as well as several variants with a different plasmid content (Table 3). These variants were made by protoplast-induced curing or isolated from cultures of *L. lactis* FM03P. Phenotypic characterisation of the variants started always with streaking the variants on M17 agar plates [67] supplemented with either 0.5% (*w*/*v*) glucose or lactose. After incubation for 2 days at 30 °C a single colony was inoculated in M17 supplemented with 0.5% lactose or glucose and grown overnight at 30 °C.

### Whole genome sequencing

#### Sequencing of FM03P

*L. lactis* FM03P was plated on M17 agar plates supplemented with 0.5% lactose (LM17) and incubated for 2 days at 30 °C. A single colony was inoculated in LM17 broth and incubated overnight at 30 °C. Subsequently, genomic DNA was extracted using the Wizard® genomic DNA purification kit (Promega, USA), sequenced using an Illumina HiSeq 2500 and a PacBio RS instrument, *do novo* assembled into contigs and scaffolds and closed by PCR and Sanger sequencing. A more detailed explanation of the method can be found in the genome announcement of *L. lactis* subsp. *lactis* biovar diacetylactis FM03 [13]. In addition to the complete chromosomal sequence, 7 plasmids were found, which were defined as complete circular contigs with a origin of replication and which could be targeted in a PCR.

#### Sequencing of FM03-V1 and the plasmids pLd8, pLd9, pLd10, pLd11 and pLd12

*L. lactis* FM03-V1, a single colony isolate of a culture of FM03P, was pre-cultured as described above. Subsequently, genomic DNA was extracted using the DNeasy Blood & Tissue kit (Qiagen, Germany) according to the manufacturer’s procedure, sequenced using an Illumina HiSeq 2500 instrument (total 2.3 million quality-filtered paired-end reads and average coverage of 211 times) and de novo assembled into contigs using VelvetOptimiser 1.1.0 (k-mer of 83). The contig sequences were compared to the obtained sequence of *L. lactis* FM03P, of which only plasmids pLd1 to pLd7 were known at that time. Unmapped contigs with a high coverage were further assembled into scaffolds and closed by PCR and Sanger sequencing. The obtained complete circular sequences are designated plasmids pLd8, pLd9, pLd10, pLd11 and pLd12.

### Protoplast-induced curing

Protoplasts of *L. lactis* FM03P were prepared in Tris-HCl magnesium chloride sucrose buffer (pH 8.0) according to Fujita et al. [68] with some modifications. Briefly, *L. lactis* was grown in LM17, harvested in mid-exponential phase by centrifugation (5 min, 6000×*g*), washed twice with 30 mM Tris-HCl buffer (pH 8.0) and resuspended in a buffer for the lysozyme treatment to degrade the cell wall (30 mM Tris-HCl, 3 mM MgCl_2_, 20% sucrose, 100–1000 μg/ml lysozyme, pH 8.0). After incubation at 37 °C for 10, 60, 120 or 180 min, protoplast were regenerated by plating appropriate dilutions of treated samples on regeneration medium (LM17 agar supplemented with 20% sucrose). After incubation at 30 °C for 48 to 72 h, regenerated colonies were picked and screened for their plasmid content.

### Screening for plasmid content

To determine the plasmid content of the variants, DNA was extracted from bacterial cultures using the DNeasy Blood & Tissue kit (Qiagen, Germany) according to the manufacturer’s procedure excluding the RNAse step. Subsequently, unique sequences of each plasmid were amplified in PCR reactions using specific primer sets (Table 4). A primer set targeting the chromosome was used as positive control for the DNA extraction. The PCR reaction mixture of 50 μl contained 1 μl purified genomic DNA, 0.2 mM dNTPs (Thermo Scientific, USA), 0.4 μM of forward and reverse primer, 5 μl of 10× Taq buffer + MgCl_2_ (Thermo Scientific, USA) and 2 U Taq polymerase (Thermo Scientific, USA). The PCR program started with an initial denaturation cycle at 94 °C for 5 min, followed by 25 cycles of 94 °C for 30 s, 58 °C for 20 s and 72 °C for 45 s and a final extension cycle at 72 °C for 7 min. For plasmids pLd7, pLd8, pLd9, pLd10, pLd11 and pLd12 annealing temperatures were increased to 68, 60, 62, 63, 63 and 60 °C, respectively. PCR amplicons were examined by gel electrophoresis in 1% agarose gels stained with SYBR®Safe DNA gel stain (Thermo Scientific, USA) and visualised under UV light (Uvitec, UK).

**Table 4:** Primer sequences used to detect plasmids in *L*. *lactis* FM03P

Target	Sequence (5′-- > 3′)		Amplicon	Tm (°C)
Chromosome	Fw	TTAATTCAACCTGGAGACACAGTCTTAG	254	65.2
	Rv	CTATCAGCGATTTCACGGAACTTAG		65.6
pLd1	Fw	GCATTGACGGCTGTTGTAAT	209	62.5
	Rv	AGCAGATTCCCGAGGATAAC		62.0
pLd2	Fw	AATGGGCCGAAGGTTCTATT	285	63.4
	Rv	CAGGAACCGATTCTCCTGTTA		62.9
pLd3	Fw	CCTCTCGCGTTCCTTGATA	417	62.9
	Rv	CCACGTAAGGGCGATTTAGT		62.7
pLd4	Fw	GCGGTAACAACATCCGTATC	508	61.8
	Rv	AGTCAGCCCAAGCGACTAAT		62.7
pLd5	Fw	AAATACAAGTGTTGAAGGCGTTG	589	63.8
	Rv	ACCTTTGTTCTCCAATTTCAGC		62.2
pLd6	Fw	TAAGTGCAACTAAAAGAAATAATAAAGTGCAA	200	65.1
	Rv	TTGCTGATGATTGTACCAGCTAAAAC		65.8
pLd7	Fw	TGGGCATCTAGATAATCTGACGACATCTGT	596	71.3
	Rv	CGACATTGACTCCCCAAAAACCAAAAATGA		75.3
pLd8	Fw	CCCAGTTGATTTAGAATTAGCTGAAGAATA	588	65.8
	Rv	AGATAGGTTGCATCCAAGATAAATTTGTTA		65.9
pLd9	Fw	TAGTCGCTGGCAAATTTTACAATCA	323	67.0
	Rv	CTTTGGGGGTTGCTTTAGAATCAAT		67.5
pLd10	Fw	ACGCTTGAACCCCATCTTGG	255	68.3
	Rv	TCGTCCCAAACGGTTTACCC		67.8
pLd11	Fw	TTCAATGAATGGCTCGGAAGAA	388	67.6
	Rv	TTTCGGCACAGGAGCAACAT		67.9
pLd12	Fw	GAAAACTAATCTAGTACAATCATCAGCAAACTT	277	65.1
	Rv	TTTTTACGAGTTATATTGTTTCTAGTCAGATTCTT		64.7

Primers were used to determine the plasmid content of different variants and to discriminate variants in competition experiments

### Phenotype testing

#### Growth in LM17

The maximum growth rate of all variants in LM17 was determined with the two-fold dilution method in a Bioscreen C as described by Biesta-Peters et al. [69]. Briefly, maximum growth rates were determined with three biological replicates and two technical replicates and performing five dilutions per replicate. A single colony was inoculated in LM17 broth and grown overnight at 30 °C. Subsequently, 500 μl of the overnight culture was transferred to 9.5 ml fresh LM17 and grown for 3 h at 30 °C to have an exponentially growing culture. The exponential culture was diluted > 1000 times in LM17 medium and two-fold dilutions were made in a 100-well Honeycomb plate, which was incubated for 24 h at 30 °C with measurements of the optical density at 600 nm every 5 min. Before each measurement, the plate was shaken for 15 s. The lowest dilution contained 10^4^ CFU/ml at the start of the incubation. The time to detect an optical density of 0.2 was determined for each dilution from which the maximum growth rate was determined as described by Biesta-Peters [69]. The maximum growth rates correspond to − 1/slope when plotting the natural logarithm of the inoculum concentration versus the time to detection. Representative wells of the highest inoculum concentration were used to compare the growth curves (Fig. 6a).

#### Growth performance experiments

The growth performance of variants 48 and 50, differing in plasmid pLd2, was assessed by sequential propagation in mixed cultures. The variants were grown overnight in M17 supplemented with 0.5% glucose (GM17) and mixed in a ratio 1:1 based on optical density measurements at 600 nm in GM17 supplemented with 0.035 mM CdCl_2_. Subsequently, the culture was incubated at 30 °C and propagated every 48 h at 1% inoculation level (100 μl added to 10 ml fresh medium) for 4 times. To determine the ratio of the variants at the end of the propagation (~ 27 generations), appropriate dilutions of samples were plated on GM17 agar and incubated for 3 days at 30 °C. Subsequently, the plasmid content was determined of approximately 20 single colonies in three steps: i) DNA extraction, ii) PCR, and iii) gel electrophoresis. DNA was extracted from single colonies by a lysis treatment followed by DNA extraction using the InstaGene™ Matrix (Bio-Rad, USA). Colonies were incubated for 30–45 min at 37 °C in 200 μl lysis buffer consisting of 20 mM Tris-HCl, 2 mM EDTA and 1 mg/ml lysozyme (pH 8.0). Subsequently, samples were centrifuged at 13800×*g* for 2 min and washed with phosphate buffer saline. The pellets were then treated with the InstaGene™ Matrix (Bio-Rad, USA) according to the manufacturer’s procedure using 100 μl matrix instead of 200 μl. PCR and gel electrophoresis were performed as explained for the screening of the plasmid content. We used pLd2 to distinguish variant 48 and 50 and pLd1 was used as positive control for a successful DNA extraction.

## Additional file

Additional file 1: **Figure S1.** Summary of sequencing attempts. **Figure S2.** PCR products confirming the presence of the 12 plasmids in *L. lactis* FM03P. **Figure S3.** Multiple sequence alignment of *repB* promoters of theta-type replication plasmids in *L. lactis* FM03P. **Figure S4.** Multiple alignment of RepB amino acid sequences of theta-type replication plasmids of *L. lactis* FM03P. (PDF 1657 kb)

## Abbreviations

AT: Acetyltransferase
bp: Base pairs
*dso*: Double-stranded origin
EPS: Exopolysaccharide
GT: Glycosyltransferase
Opp: Oligopeptide permease
Pfam: Protein family
PLD: Phospholipase D
PTS: Phosphotransferase system
RCR: Rolling-circle replication
TRD: Target recognition domain
