# Supplementary material for: Large plasmidome of dairy Lactococcus lactis subsp. lactis biovar diacetylactis FM03P encodes technological functions and appears highly unstable
Source: BMC Genomics. 2018 Aug 17;19:620. doi: 10.1186/s12864-018-5005-2 (PMC6098607; doi:10.1186/s12864-018-5005-2)
Supplement: Supplementary file 1 — Figure S1. Summary of sequencing attempts. Figure S2. PCR products confirming the presence of the 12 plasmids in L. lactis FM03P. Figure S3. Multiple sequence alignment of repB promoters of theta-type replication plasmids in L. lactis FM03P. Figure S4. Multiple alignment of RepB amino acid sequences of theta-type replication plasmids of L. lactis FM03P. (PDF 1657 kb) [file 12864_2018_5005_MOESM1_ESM.pdf]

Additional file 1: Supplementary figures

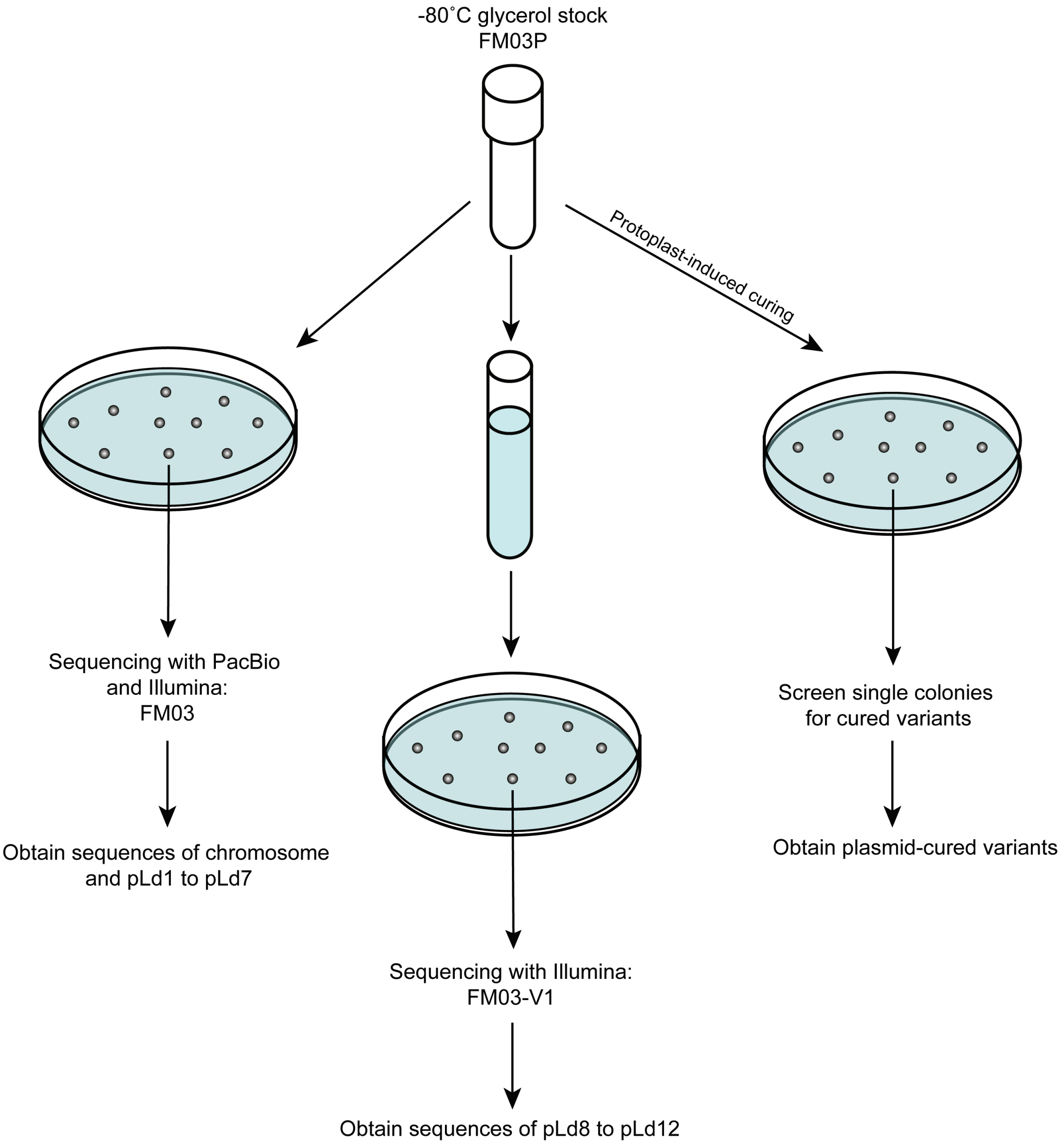

Supplementary Figure S1: Summary of the sequencing attempts.

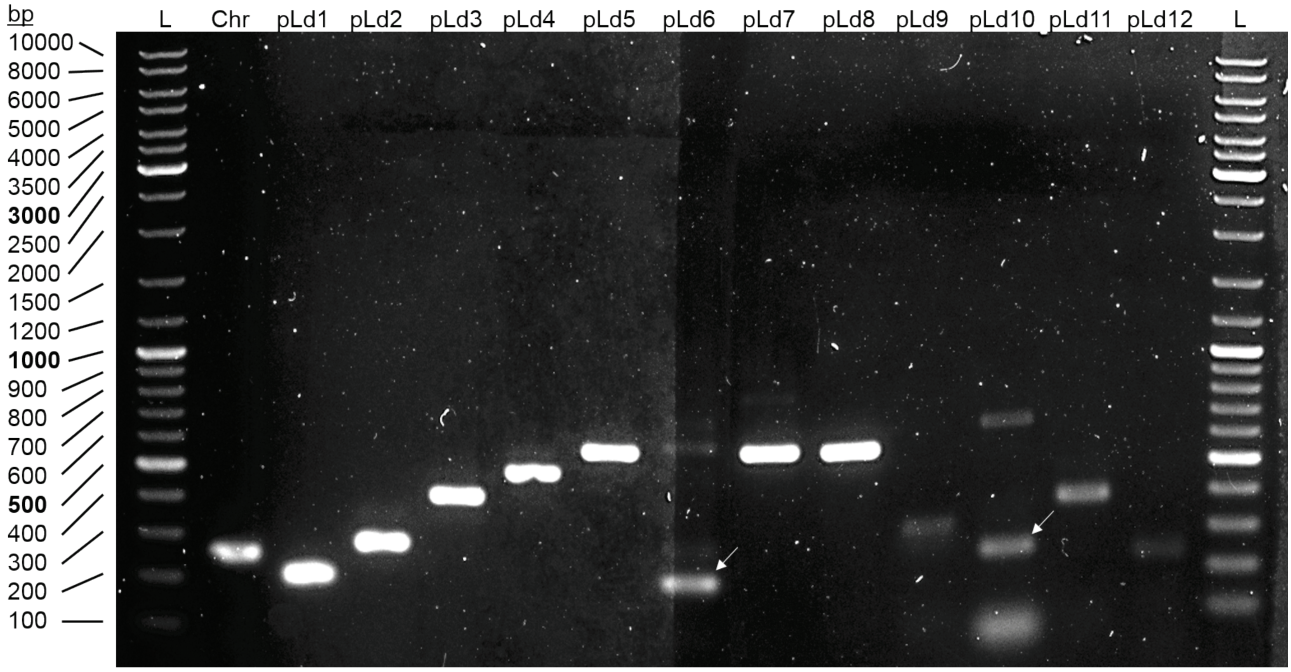

Supplementary Figure S2: PCR products confirming the presence of the 12 plasmids in *L. lactis* FM03P. PCRs were performed using the primers listed in Table 4. In case of multiple bands, white arrows indicate the band with the expected size as listed in Table 4. L: DNA Ladder mix #SM1173 (Thermo Scientific, USA).

```
                                               AT-rich
pLd1          ------TTTTAAATTT----TTGAAAAAAATAAAAAA-----------AGGCG-AAGCC--TATTATATATTTATCATATATATTT
pLd4          TTTTGACCCTGATTTTAAGATTGAAAAAAATAAAAAATAGACTTGCCTTAGCA-AGTCC--TTTTATTAATTTATCTTATATATTT
pLd2          ------TTTTAAATTT----TTGAAAAAAATAAAAAA----------TAGGCG-AAGCCTATA-TATATATTTATCTTATATATTT
pLd9          ------TTTTAAATTT----TTGAAAAAAATAAAAAA----------TAGGCG-AAGCCTATA-TATATATTTATCTTATATATTT
pLd7          -----ATTTTAAATTT----TTGAAAAAAATAAAAAA-----------AGGCG-AAGCC--TA-TATTAATTTATCATATATATTT
pLd10_repB2   TTTTGATTTTAAATTT----TTGAAAAAAATAAAAAA-----------AGGCG-AAGCC--TA-TATTAATTTATCATATATATTT
pLd5          -TTTATTTTTACATTT----TTGAGAAAAATAAAAAA----------TAGGCG-AAGCC--TATTATATATTTATCTTATATATTT
pLd10_repB1   -TTTGATTTTAAGATT----TTGAAACGGAGTGAAAA----------AAGGCG-AAGCC--TA-TATATATTTATCATATATATTT
pLd11         -CTTGATTTTAAGATT----TTTAAACGGAGTGAAAA----------AAGGCG-AAGCC--TA-TTATTATTTATCTTATATATTT
pLd8_repB2    --------------TT----TTAAAAGGTTATCAAAA----------AAGGCGTTAGCC--TA-TTTATATTTATCATATATATTT
pLd6          TTTTCTTTTTAATTCTTTG-TTGAAAAGTTTTA---------------------AAGCC--TA-TATTAATTTATCTTATATATTT
pLd12         TTTTCTTTTTAATTCTTTG-TTGAAAAGTTTTA---------------------AAGCC--TA-TATTAATTTATATTATATATTT
pLd8_repB1    -------------TAT----TTTAAAAAAAGAAAATTGGGACTC-CTTAG----AGTCCCTTTTAATTATTTTATATTATATATTT

                         AT-rich                                     22-bp DR
pLd1          TAATCTTTTGTTCTTTTGCGTGAAAAAAAAGGCAGTGTTTTCGCTAGTTATAGAAATTAAACAGTCACAAAAATCGATGTATAGAG
pLd4          TAATCTTTTGTTCTTTTGTGTGGTTTAAAAGTCAATGTTTTCAAAAATTTACAGATTTATAGCGGTAGAAAAAACTGTGTATAGCG
pLd2          TAATCTTTTATTCTTTTGCGTCAAAAAAAAATCAATATTTTCAAGGCTTTATAGAATTATATACCAACAAAAAACTGTGTATATAC
pLd9          TAATCTTTTATTCTTTTGCGTCAAAAAAAAATCAATATTTTCAAGGCTTTATAGAATTATATACCAACAAAAAACTGTGTATATAC
pLd7          TAATCTTTTCTTCTTTTGCGTCAAAAAAAAGTGAGTGTTTTCAAGGGGTTAAAGAATAATATGTGGAGAAAAAACTGTTTATATAT
pLd10_repB2   TAATCTTTTCTTCTTTTGCGTCAAAAAAAAGTGAGTATTTTTAAGGGGTTACAGAATAATATAGCATAAAAAAACTGTGTATATAG
pLd5          TAATCTTTTA-TCTTTTGCGTGAAAAAAAAGTCAGTATTTATAGGCTTATACAAATTTTTATAGCAAGAAAAAACTATGTATATAG
pLd10_repB1   TAATCTTTTGTTCTTTTGCGTGAGGAAAAAGTCAGTTTTTTCAAGGGGATACAGATTTTTTAGGAGAGAAAAAACTATGTATAAGG
pLd11         TAATCTTTTGTTCTTTTGCGTGAAAAAAAAGTCAGTGTTTGCAAGGTTAAATAGATTTATACCGAGAGAAAAAACAATGTATACCG
pLd8_repB2    TAATCTTTTGTTCTTTTACGTGGTGAAAAAGTCAATGTTTCCGCCAATTATATAAATTATACCAAGACAAAAAACAATGTATACCA
pLd6          TAATCTTTTA-TCTTTTGCGTGAAAAAAAAGTCAGTGTTTTCAAGGGTATGCAGAATTTAATACGTAAAAAAAACTGTGTATACAC
pLd12         TAATCTTTTA-TCTTTTGCGTGAAAAAAAAGTCAGTGTTTCCAAGGGTATGCAGAATTTAATACGTAAAAAAAACTATGTACACAC
pLd8_repB1    T-GTCTTTTGTTCTTTTGCGAAAAAAAAAAATCTAGTGTTTGCAAGGGGTAACAGGATTATAGTCCTACAAAAAACTGTGCATAGTC

                  22-bp DR           22-bp DR            IRa                      IRa       -10
pLd1          TCACAAAAATCGATGTATAGAGTCACAAAAATCGATGTACACAGTACGA----CTTTTGTATTTGTGTGCTGTATATAGTATAAT
pLd4          GTAGAAAAAACTGTGTATAGCGGTAGAAAAAACTGTGTATAGCGGTAGAAAA-TTATTTTATTATTACCGCTATTGTGATATAAT
pLd2          CAACAAAAAACTGTGCATACACCAACAAAAAACTGTGCATATACCAACTTCT-TTGTTTGTTTCGTTGGTATATAATGATATAAT
pLd9          CAACAAAAAACTGTGCATACACCAACAAAAAACTGTGCATATACCAACTTCT-TTGTTTGTTTCGTTGGTATATAATGATATAAT
pLd7          GGAGAAAAAACTGTTTATATATGGAGAAAAAACTGTTTATACATGGAGAATG-TCTATTATCTTCTCCATGTATTATGGTATAAT
pLd10_repB2   CATAAAAAAACTGTGTATATAGCATAAAAAAACTGTGTATATAGCATAAAAA-AATCATCAGTTTATGCTATATTATGATATAAT
pLd5          CAAGAAAAAACTATGTATATAGCAAGAAAAAACTATGTATATAGCAAGAAAA-AACTATAAAAACTTGCTGTATTATGATATAAT
pLd10_repB1   AGAGAAAAAACTATGTATAAGGAGAGAAAAAACTATGTATAAGGAGAGAAAA-ATTTATCGTTCCTCTCTTTATTATGATATAAT
pLd11         AGAGAAAAAACAATGTATACCGAGAGAAAAAACAATGTATACCGAGAGAAAATAAATGTTTTTCTCTCGGTATTTTGATATAAT
pLd8_repB2    AGACAAAAAACAATGTATACCAAGACAAAAAACAATGTATACCAAGACAATT-TATTAAAAAATGTCTTGGTAT-GTGATATAAT
pLd6          GTAAAAAAAACTGTGTACACGCGTAAAAAAAACTGTGTACACACGTAAAAA--ACTTTATTAGTTTACGTGTTTTTGATATAAT
pLd12         GTAAAAAAAACTATGTACACACGTAAAAAAAACTATGTACACACGTAAAAA---CTTTATTAGTTTACGTGTTTTTGATATAAT
pLd8_repB1    CTACAAAAAACTGTGTATAGTCCTACAAAAAACTGTGTATAGCCCTACAAGT-TATTTGTGTTTGTAGGTGTTTCGTGTTATTAT

                   IRb                        IRb                          RBS           start
pLd1          -AAAAGCATAGAGAAAACTCACTATGAAATGACTTTCTCTATGCTACTACTAAAACACG--CAAAGGAGCGTATTTATACTATG
pLd4          AAAAAGCATAGAGAAATTTCACGACTAAATGACTTTCTCTATGCTACTCTCAAA-CACG--CAAAGGAGCGTATTTATATTATG
pLd2          -AAAAGCATGAAGAA-TCTCTCTACGAAAAGTGTTTCTTCATGCTTATCTAAACTCACTCACAAAGGAGCAGTTTT---CTATG
pLd9          -AAAAGCATGAAGAA-TCTCTCTACGAAAAGTGTTTCTTCATGCTTATCTAAACTCACTCACAAAGGAGCAGTTTT---CTATG
pLd7          -AAAAGCATGAAGAA-ACACTTTTCGTCGAGAGAATCTTCATGCGTATCTAAAAACACT--CAAAGGAGCGTATTT---CTATG
pLd10_repB2   -AAAAGTATGAAGAACAAACTTTTGAACGAGAATTTCTTCATACTTACTTATGAACACG--CAGAGGAGCGTATCT---TTATG
pLd5          -AAAAGCATAGAGAAAATTCACGACGAAATGA-CTTCTCTATGCTTAGCCAAAATTACTC-ATAAGGAGCAACTTC---TCATG
pLd10_repB1   -AAAAGCATGAAGAAATTCCGTCTAAAGAATT-TTTCTTCATGCTTAACCAAAATTACTC-ATAAGGAGCAACTTC---TCATG
pLd11         -AAAAGCATGAAGAAATTCCGTCCAAAGATAT-TTTCTTCATGCTTAACTAAAATTACTC-ATAAGGAGCAACTTC---TCATG
pLd8_repB2    -AAAAGCATAGAGAAATCAGACGGAAAAATCAGTTTCTCTATGCTTAACCAAAATTACTC-ATAAGGAGCAACTTC---TCATG
pLd6          -AAAAACATAGAGAAATTGACTCGCTAAAGATTTTTCTCTATGCCTATTTAAAAACACTCACAAAGGAGTA--TTA---CTATG
pLd12         -AAAAACATAGAGAAATTCACTCGGAAAAATGTTTTCTCTATGCCTATCTAAAATCACTCACAAAGGAGTA--TTT---ACATG
pLd8_repB1    ---------------------------------------------TTATTTAAATCAT---AAAAGGAGTGGATT------ATG
```

Supplementary Figure S3: Multiple sequence alignment of *repB* promoters of theta-type replication plasmids in *L. lactis* FM03P. Indicated are the AT-rich regions, the 22-bp direct repeats and in bold the inverted repeats IRa and IRb. The extended -10 promoter site and ribosome binding site (RBS) are shaded. The ATG start codon of *repB* is shown at the end of the alignment in bold.

```
pLd1          M-IIP------EKQNKQKQVLTLNELEKRKVVEHNALIQSVAKMQKTALKMFELAVSCIDTEEPPKNNTVYLLKSELFKFF
pLd2          MSSIS------KNEPNQKQVQTLNELSKRKVVEHNSLITSIAKMDKTPLKMFELAVSCINTEEPPKDHTVYLSKEELFAFF
pLd9          MSSIS------KNEPNQKQVQTLNELSKRKVVEHNSLITSIAKMDKTPLKMFELAVSCINTEEPPKDHTVYLSKEELFAFF
pLd4          M-IIP------EKNKNQKQLLSLNELSKRKVVEHNSLITSIAKMDKTPLKMFELAVSCINTEEPPKDNTVYLSKRDLFAFF
pLd7          MSSIP------KNEQNQKQVQILNELSKRKVVEHNSLITSIAKMDKTPLKMFELAVSCIDTEEPPKDHTVYLSKEELFTFF
pLd10_RepB2   MSIIT------EFEKNQKQVKALNELSKRKVVEHNSLITSIAKMDKTPLKMFELAVSCINTEAPPKDHTVYLSKTELFAFF
pLd6          MPSIA------EKQENQKQVLTLNELSKRKVVEHNSLITSIAKMDKTPLKMFELAVSCINTEEPPKDNAVYLSKEELFSFF
pLd12         MPIIT------EKQENKKQVLTLNELSKRKVVEHNSLITSIAKMDKTPLKMFELAVSCINTEEPPKDNAVYLSKEELFSFF
pLd8_RepB2    MEIISK----NERNNNERTVCSLKEIEKRKIVEHNDLITSVAKMDKVPLKIFELAVSCIDTENLPKDNIIYLSKAELFTFF
pLd10_RepB1   MEIITK----NDRNSNERIVCSLKEIEKRKIVEHNDLITSIAKMDKVPLKIFELAVSCIDTENLPKENTIYLSKSELFSFF
pLd11         MEIITK----NDRNNNERTVCSLKEIEKRKIVEHNDLITSVAKMDKVPLKIFELAVSCIDTENLPKDNTIYLSKEELFTFF
pLd5          MEIIAKKYPYNNYKDNERTVCSLKEIEKRKIVEHNDLITSVAKMDKVPLKIFELAVSLIDTDNPPKNNTIYLSKTELFAFF
pLd8_RepB1    MQKID------TGERNKQ----IQEISSRKVAEHNDLISSVAKMDKTPLKMFELAVSCIDTDAPPKDNIVFLSKKELFTFF

pLd1          EVSSSSKHSQFKEAVNYMQKQAFFNIKADK--KLGIEYESIVPIPYVKWNDYNDEVTIRFDQAIMPYLIDLKAEFTQYKIS
pLd2          KVSDNDKHSRFKQAVENMQKQAFFQIKEEV--GKGFKFRSIVPIPYVEWTDYHDDVKIEFHREIMPYLINLKQNFTQHALS
pLd9          KVSDNDKHSRFKQAVENMQKQAFFQIKEEV--GKGFKFRSIVPIPYVEWTDYHDDVKIEFHREIMPYLINLKQNFTQHALS
pLd4          KVSDNDKHRRFKEAVEKMQKQAFFQIKEEE--GKGFKFKSIVPIPYVEWTDYHDEVKIEFHREIMPYLINLKKNFTQHALS
pLd7          KVSDNDKHSRFKQAVENMQKQAFFKIKEKK--EHGFEFENIVPIPYVKWTDYHDEVTIRFSPEIMPYLINLKKNFTQHALS
pLd10_RepB2   KVSDNDKHSRFKQAVENMQKQAFFKIQEKK--EYGFEFENIVPIPYVKWADYHDEVTIRFSPEIMPYLINLKQNFTQHALS
pLd6          KVSDNDKHSRFKQAVAKMQEQAFFEIKEEK--NKGFKFRRILPIPTVEWTDYDDKVMIRFNQDIMPYLIELKQNFTKYALS
pLd12         KVDDSNKHTRFKQAIAKMQEQAFFEIKEEK--NKGFKFRRILPIPTVEWTDYDDKVMIRFNQDIIPYLIELKQNFTKYALS
pLd8_RepB2    DVSDNGRHTRFKEAIEKMQKQAFFEIKEVK--GKGYNVKSIVPIPYVEWNSYNDMVTLQFQPQIMPYLIDLKKNFTQYALS
pLd10_RepB1   KVDDSNKHHRFKEAIKVMQKEAFFEIREEA--GKGFKFKSIVPIPYVEWTDYHDEVKIEFHREIMPYLIELKKNFTQYALS
pLd11         DVSDNGKHTRFKEAISKMQEQAFFEVREAK--NKGFKFRRILPIPTVEWTDYDDKVMIRFNQDIMPYLIELKQNFTQYALS
pLd5          KVNDSNKHSRFKEAIMTLHQQSVFEIREMNVHKNKFEYRVISPLEETTWNDYNDIISMTFTKSIMPYLIELKTKFTQYALS
pLd8_RepB1    DVSDNDKHRRFKEAVEKMQEQAFFRIKEKK--NRGFKFKRIVPIPYVEWNDYNDKVLIRFDQAIMPYLIDLKNNFTQYAIS

pLd1         ELQKLNSKYSIILYRWLSMNYNQYEHYSVKGGRRVEQVESYRNPSIKVKELREITDTINEHQHFPHFETRVLKKAIEEINAH
pLd2         DIAELNSKYSIILYRWLSMNYNQYEHYSYKGGRREEQVEAYRNPTISMRELREMTDTVDEYPRFDRLEHRVLKEPIEEINEN
pLd9         DIAELNSKYSIILYRWLSMNYNQYEHYSYKGGRREEQVEAYRNPTISMRELREMTDTVDEYPRFDRLEHRVLKEPIEEINEN
pLd4         DIAELNSKYSIILYRWLSMQYNQYEHYSVKGGRRAEQVEAYRNPSITVKELRTITDTVNEYKDMNNFTKFVLDKPLKEINTH
pLd7         DISELNSKHSIILYRWLSMNYNQYEHYSYKGGRREEQVEAYRNPSISIRELREMTDIVNEYKLFADLEKWILKKPLEEINDH
pLd10_RepB2  DIAELNSKYSIILYRWLSMNYNQYEHYSAKGGRREEQVETYRNPSISIRELREMTDTMKDYPRFQSLESYIIKNSLKEINEH
pLd6         EIMELNSKYSIILYKWLSMNYNQYEHYSNKGGRRAEQVESYRNPSISVKELRTITDTVNEYKETYHFFRYIVENSLKEINAH
pLd12        EIMELNSKYSIILYKWLSMQYNQYEHYSNKGGRRAEQVENYRNPSISVKELRTITDTVNEYKETYHFFRYIVENSLKEINAH
pLd8_RepB2   DVMELNSKYSIILYKWLSMNYNQYEHYSVKGGRRTEQVESYRNPSIKVSELRIITDTMNEYKNFTDFNRRVLKEPLEEINAH
pLd10_RepB1  DVMELNSKYSIILYKWLSMNYNQYEHYSAKGGRRVNQVESYRNPSITVNELREITDTIHEYKRMQDFTRWTLEKPLEEINAH
pLd11        DLIDLNSKYSIILYKWFSMSYNQYEHYRAKGGRRAEQVEAYRNPSITVKELRIITDTTNEYKNFTDFSRWILNKPLAEINAH
pLd5         DLVDLNSKYSIILYKLLSMNYNQYEHYSNKGGRRAEQVEAYRNPSIPVKELRVITDTVNDYVRFQSLETWVLKKPLEEINAH
pLd8_RepB1   DIMELNSKYSIILYKWFSMSYNQFEHYQYKPNRTKKQLEDYKSPRIIISDLRELTDTVDDYSRFDNFEKRVIKDAIKEINSF

pLd1         TSFNVTYEKKKAGRSIDSIVFHIEKKRMADDNSYKLGDKDYQDDKKQKSRNEADLLKQAMESKYTRLLSENFLIGMNDIMDT
pLd2         TSFNVTYDKIKKGRSIDSIVFHITKKRRADDNSYKLEDKDYQSDKEEKSRNEADLLKQAMESKYTRLLIENFLLSPLEMTDT
pLd9         TSFNVTYDKIKKGRSIDSIVFHITKKRRADDNSYKLEDKDYQSDKEEKSRNEADLLKQAMESKYTRLLIENFLLSPLEMTDT
pLd4         TTFNVSYDKIKKGRSIDSIVFHIEKKRCADDNSYKLGDKDYQADKKQKSRNEADLLKQAMESKYTRLLSENFLIGMNDIMDT
pLd7         TSFTVTYDKVKKGRSIDSIVFHITKKRRADDNSYKLEDKVYQEDKARKAETEDMLTVQALKSPYTKLLMEHFLLSYLDLTDT
pLd10_RepB2  TSFKVTYEKVKKGRSINSIVFHITKKRRADDNSYKLEDKVYQKAKVQKEQKENLLYAEAMQSKYTKLLLEHFLLSPYEMTNP
pLd6         TSFNVSYEKIKKGRSIDSIVFHIEKKRKADDNSYKLEDQAYIEGKKAKEETEKDLYTEAMQSPYTKLLSENMLLFPNDFMDI
pLd12        TSFNVSYEKIKKGRSIDSIVFHIEKKRKADDNSYKLEDQAYIEGKKAKEETEKDLYTESMQSPYTKLLSENMLLFPNDFMDI
pLd8_RepB2   THFNVFYDKIKAGRSIDSIQFHIEKKRRADDNSYKLEDKDYQSDKEEKSRNEADLLKQAMESKYTRLLIENFLLFPLEMTDT
pLd10_RepB1  TSFNVTYEKVKKGRSIDSVVFHIERKRSADDNSYKLENTAYQEGNKVKEETEDKLAIEAMKSKYTKLLIENFLLSPLEMTDT
pLd11        TSFNVTYDKIKKGRSIDSIVFHIEKKRKADDNSYKLEDTAYQEDKARKAETEDKLAIEAMKSKYTTLLLENMLLSPFEMQDT
pLd5         TSFNVTYDKIKKGRSIDSIVFHIEKKRMADDNSYKLDNRAYQEDKKQKETDNRLLAGEALENKYTKLLIEHFLLSPYDMTDT
pLd8_RepB1   THFNVEYKKIKKGRSIDSIQFHIVKKANWKDENYKRNDVQAQLTEEQNQAQNQVNYAVAVANPFTMKLINSSLLYATDIANQ

pLd1         ATMVGLQKNVYPLYDELKELRGLNGVKDHLSYVASKKEAYS--KRNVAKYLKKAIEQYLPT-VKRQDLNHE-------------
pLd2         ALMAGLQKNVYPLYDELKELRGLNGVKDHLSYISSKKEAYS--KRNVAKYLKKAIEQYLPT-VKRQDLNHE-------------
pLd9         ALMAGLQKNVYPLYDELKELRGLNGVKDHLSYISSKKEAYS--KRNVAKYLKKAIEQYLPT-VKRQDLNHE-------------
pLd4         ATMVGLQKNVYPLYDELKELRGLNGVKDHLSYVASKKEAYS--KRNVAKYLKKAIEQYLPT-VKRQNL----------------
pLd7         KILSGLQAHVYPLYDELKDLRGLNGVKDHLSYVRAKREDYS--KKNITKYLKKAIEQYLPT-VKRQDL----------------
pLd10_RepB2  ATMAGLQRNVYPKYDELKDLMGIDGVKKHLSYIYDKQEPYS--KGNIAKYLKKAIEQYLPT-VKRRGL----------------
pLd6         KTMAGLQKNVYPLYDELKALRGLNGVKGHLSYVSSKQEAYS--KRNIAKYLKKAIEHYLPT-VKRQDL----------------
pLd12        KTMAGLQKNVYPLYDELKELKGLNGVKDHLSYVSSKQEAYS--KRNIAKYLKKAIEHYLPT-VKRQDL----------------
pLd8_RepB2   ALMAGLQKNVYPLYDELKELRGLNGVKDHLFYVSSKQEAYS--KRNVAKYLKKAIEQYLPT-VKRQEFVMRSLK----------
pLd10_RepB1  ATMAGLQAHVYPLYDELKELRGLNGVKDHLSYVRAKQEDYS--KKNIARYLKKAIEQYLPT-VKRRGL----------------
pLd11        KIMAGLQVHVYPLYDELKALRGLKGVKDHLSYVASKQEAYS--KRNVAKYLKKAIEQYLPT-VKRQDLNHE-------------
pLd5         ATMAGLQKNVYPLYDELKDLRGLNGVKDHLSYVASKKEAYS--KRNVAKYLKKAIEQYLPT-VKLQDLEQPERAKVRGKGASHE
pLd8_RepB1   DTILELAESVYPVYDKLVKELGEDALETHMDYVRRKMVDYSNDKKNIVKYLSISAKQYLNSRLSKQQMKE-------------
```

Supplementary Figure S4: Multiple alignment of RepB amino acid sequences of theta-type replication plasmids of *L. lactis* FM03P. Amino acids are shaded black if they follow the consensus sequence (same amino acid in at least half of the sequences). Amino acids are shaded grey if they are similar to the consensus.
